# Effect of ZrC Formation on Graphitization of Carbon Phase in Polymer Derived ZrC–C Ceramics

**DOI:** 10.3390/ma12244153

**Published:** 2019-12-11

**Authors:** Changqing Liu, Luyue Zhang, Xiaoxiao Yuan, Xu Li, Yuanting Wu, Xiufeng Wang

**Affiliations:** School of Material Science and Engineering, Shaanxi Key Laboratory of Green Preparation and Functionalization for Inorganic Materials, Shaanxi University of Science & Technology, Xi’an 710021, China

**Keywords:** carbothermal reduction, ZrC, graphitization, carbon

## Abstract

Graphitization degree of carbon matrix in ZrC-modified carbon composites is crucial to mechanical and ablation properties of the materials. In order to investigate the effect of ZrC formation on graphitization of the carbon matrix, microstructure of the carbon phase was investigated by X-ray (XRD), Raman, and X-ray photoelectron spectroscopy (XPS) analysis of the ceramic products obtained from zirconium containing polymer precursors at different pyrolysis temperatures. Compared with pure carbon phase, significant increase of average crystal thickness and microcrystalline planar size was observed in the carbon phase of the ZrC–C ceramics, together with the decrease of interlayer spacing and integrated intensity ratio of D peak to the G peak, indicating a significantly increased graphitization degree during the formation of ZrC. With the increasing ZrC content, amorphous (A) carbon was reduced remarkably, while turbostratic (T) component and graphitic (G) carbon components were increased, showing a slight higher graphitization degree. Moreover, the formation of ZrC was the key “ice breaking” step to decrease the defects and improve structure order of the carbon matrix. And the graphitization was dramatically enhanced during carbothermal reduction to create ZrC by breaking the carbon structure. Furthermore, coarsening and aggregation of ZrC particles as a result of high-temperature heat-treatment at 2500 °C and a high content of ZrC exhibit some negative influences on the structure of the carbon phase in ZrC–C ceramics.

## 1. Introduction

Carbon materials, including graphite and carbon/carbon (C/C) composites have been widely used in astronautic and aerospace industries because of their excellent thermo-physical and mechanical performance at high temperature [1,2]. However, the key drawback of carbon materials is their susceptibility to oxidation above 500 °C, which has seriously limited their further application. Recently, researches have shown that the ablation and oxidation resistance of the carbon materials can be effectively improved by the addition of ultra-high temperature ceramics (UHTCs) [3], such as transition-metal carbides and diborides (ZrC, ZrB_2_, HfC, HfB_2_, and TaC, etc.).

Among UHTCs family, ZrC is regarded as one of the most promising ceramics, owing to its high melting temperature (3540 °C), relatively low density (6.7 g/cm^3^), great hardness (25.5 GPa), good chemical inertness, and excellent mechanical stability [4]. Additionally, its refractory oxide (ZrO_2_) formed in working condition can effectively reduce the diffusion of oxidizing species into the composites [5]. Moreover, researchers have discovered that ZrC could contribute to graphitization of carbon at high temperatures [6,7]. As the graphitization degree is one crucial structural parameter for carbon materials, the physical and chemical characteristics of the carbon phase, such as thermal conductivity, strength and modulus, thermal shock resistance, and electrical resistivity are greatly affected by its graphitization degree [8,9]. Therefore, some efforts have been attempted to investigate the catalytic graphitization role of ZrC by analyzing the microstructure of the carbon phase [10,11,12]. Nevertheless, there is no report concerning the influence of ZrC formation on the graphitization process of the carbon matrix.

For the formation of ZrC, carbothermal reduction reaction between ZrO_2_ and carbon matrix is the most common approach, which is also a frequently used method for the introduction of ZrC into C/C composites [13,14]. During this process, microstructure of carbon phase can be affected, which would seriously influence the graphitization behavior of the carbon matrix [15,16,17]. Especially, the pyrolysis mechanism of carbon from the ceramic precursor is yet to be reported.

To study the underlying factors on the graphitization process during the formation of carbide, zirconium containing polymer precursor was synthesized, and ZrC–C ceramics with various zirconium contents were fabricated by pyrolyzing the precursors at different temperatures. The graphitization process of the carbon phases in the obtained ZrC–C ceramics was investigated. The effect of the carbide formation on the graphitization of the pyrolysis carbon was discussed in detail.

## 2. Experimental Procedure

### 2.1. Preparation of Samples

Zirconium containing polymer (PZC) precursors with different zirconium contents were synthesized by adjusting the amount of added zirconium oxychloride (ZrOCl_2_·8H_2_O) in a mixture of ethanol, acetylacetone, and H_2_O_2_ with a volume ratio of 20:3:1 to prepare a zirconium-containing solution. The as-prepared solution was added into the hydroxymethyl phenol liquid which was synthesized from the condensation reaction between phenol and paraformaldehyde using NaOH as the catalyst with a molar ratio of 1:1.3:0.1. The reaction occurred in a three-necked flask and heated at 80 °C for 1–1.5 h in water bath, followed by heating at 90 °C for another 1–2 h to obtain a red-brown PZC precursor. The weight of ZrOCl_2_·8H_2_O in the solution was 0, 5, 10, and 15 g, respectively. The obtained precursors are abbreviated as PZC-0, PZC-1, PZC-2, and PZC-3, respectively. Detailed synthesis process and mechanism for the preparation of PZC can be found elsewhere [7]. Then the samples were placed in a graphite crucible and heated from room temperature to 1200, 1500, 1800, 2000, and 2500 °C, respectively, at a heating rate of 5 °C/min in pure argon. The holding time of the heat treatment was 2 h, and then the samples were naturally cooled to room temperature with the protection of argon. To facilitate the study, the obtained carbon phase in PZC-derived ZrC–C ceramics is named as PZC derived carbon.

### 2.2. Characterization

Microstructure of the PZC precursors was analyzed using Fourier transform infrared spectroscopy (FTIR, Vector-22, Bruker, Bremen, Germany). Elemental analysis of PZC precursors was performed by X-ray photoelectron spectroscopy (XPS, PHI-5400, PE, USA). C_1s_ XPS fitting spectra of the carbon was obtained by curve-fitting of C_1s_ multiplex spectra in a Casaxps software (version 2.3.16 PR 1.6, Casa Software) by using the function of “fit component.” Relative content of each component for the carbon phase were obtained by finishing the component fitting process in the software. Chemical states of each component were determined on the basis of references and the data provided from the software.

Moreover, the ceramic composition of the PZC derived ceramics was determined by an energy dispersive spectrometer (EDS, AMRAY, USA).

Composition and crystallinity of the obtained ceramics were recorded using an X-ray diffraction (XRD, X’pert Pro MPD, Bruker, Bremen, Germany) with as anode material of Cu. The interlayer spacing (*d*_002_) and the crystal thickness (*L*_c_) of the carbon phase were calculated using the following equations [18], respectively:

(1) $$d_{002}=\lambda /\left(2\sin\theta \right)$$

(2) $${L_{c}=K}_{2}\lambda /B(002)\cos\theta$$

where *λ* = 0.1541 nm, apparatus constant *K*_2_ is 0.94, and *B* is the half-value width in radians of the X-ray diffraction intensity. 

Separation of the (002) diffraction profiles of the carbons into their components, including amorphous (A) component, turbostratic (T) component, and graphitic (G) component, was conducted using origin software with the function of peak analyzer.

Structure order of the obtained ceramics was analyzed by a Raman spectrometer (Raman, Renishaw, United Kingdom). The microcrystalline planar size (*L*_a_) and the integrated intensity ratio of D peak to the G peak (R) were calculated as follows [19]:

(3) $$L_{a}=4.35R^{-1}$$

(4) $$R=I_{D}/I_{G}$$

where *I*_D_ represents the intensity of the peak positioned at about 1350 cm^−1^ representing the short-range turbostratic structures, and *I*_G_ is the peak intensity of about 1590 cm^−1^ corresponding to the normal graphite structure.

## 3. Results and Discussion

### 3.1. Characterization of As-Prepared PZC

**Figure 1:**
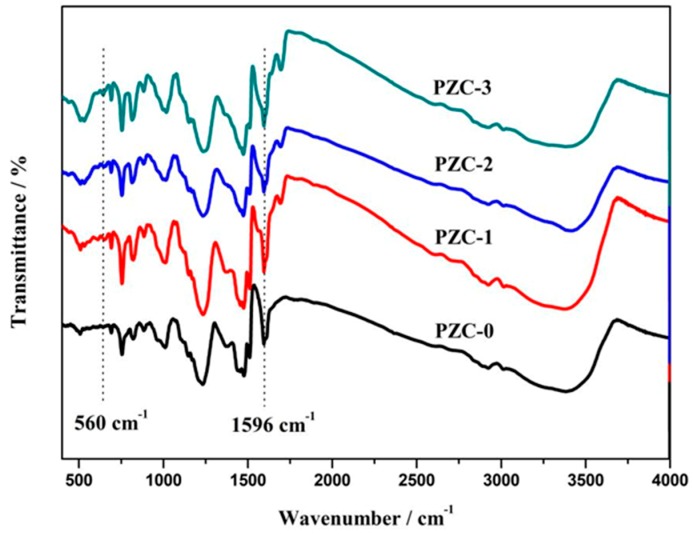
Fourier transform infrared (FTIR) spectra of as-prepared zirconium-containing polymer (PZC) precursors.

Figure 1 shows the FTIR spectra of as-prepared PZC precursors. The peaks in the spectra are assigned as follows [20,21,22]: 3343 cm^−1^ (-OH stretch in phenolic ring), 1597–1506 cm^−1^ (C=C stretch in phenolic ring), 1477–1375 cm^−1^ (-CH_2_-scissor vibration), 1450 cm^−1^ (C=O stretch), 1238 cm^−1^ (C–O stretch), 1156 cm^−1^ (CH_2_–O–CH_2_ stretch), 1024 cm^−1^ (C–O stretch in hydroxymethyl), 888–757 cm^−1^ (C–H stretch in phenolic ring), and 500–700 cm^−1^ (Zr–O vibration). The intensity ratio of peaks at 560 cm^−1^ (Zr–O) to that at 1596 cm^−1^ (C=C) is donated as *A(Zr–O)/A(C=C)*. Since C=C bond was not involved in the chemical reactions during the whole process, the value of *A(Zr–O)/A(C=C)* indicates the relative contents of the Zr–O bonds in the system. The corresponding result is listed in Table 1. It shows that *A(Zr–O)/A(C=C)* increases with the increasing ZrOCl_2_·8H_2_O contents in feed, indicating that the zirconium content increases from PZC-0 to PZC-3 as expected.

**Figure 2:**
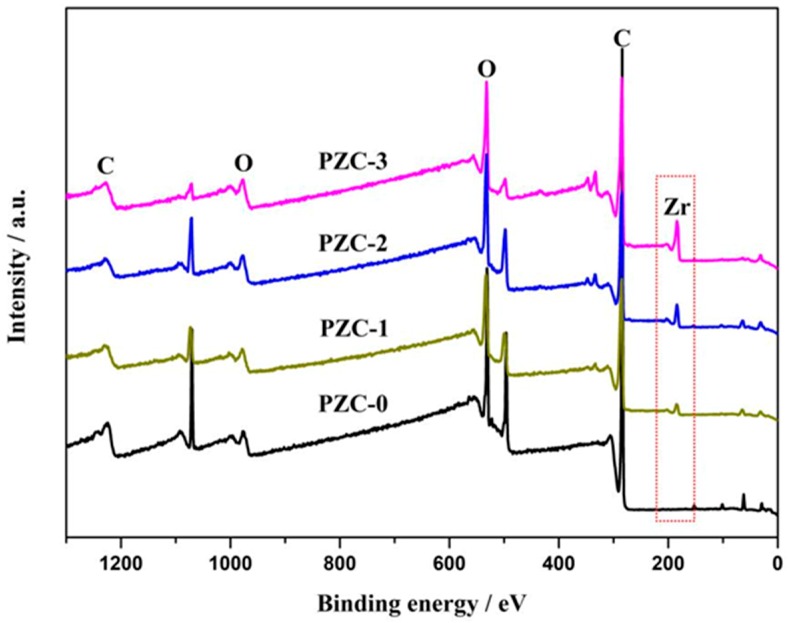
X-ray photoelectron spectroscopy (XPS) survey spectra of as-prepared PZC precursors.

To further confirm the composition of the PZC precursor, XPS analysis was applied and the results are shown in Figure 2. From the survey spectra of the samples, Zr_3d_ peak at 183 eV, C_1s_ peak at around 285.5 eV, and O_1s_ peak at 532.3 eV can be observed, indicating the as-prepared precursor consists of Zr, O, C, and undetected H. In addition, the intensity of the Zr_3d_ peak increases from PZC-0 to PZC-3, suggesting an increase of zirconium content in the precursor. As identified from the XPS analysis, mass fraction of the zirconium in sample PZC-*x* (*x* = 0, 1, 2, 3) is 0 wt.%, 5 wt.%, 10 wt.%, and 16 wt.%, respectively. For the derived ZrC–C ceramics, the zirconium contents identified from the EDS analysis are 0 wt.%, 11 wt.%, 18 wt.%, and 27 wt.%, respectively, as provided in Table 1.

### 3.2. XPS Analysis of PZC-Derived Carbons

**Figure 3:**
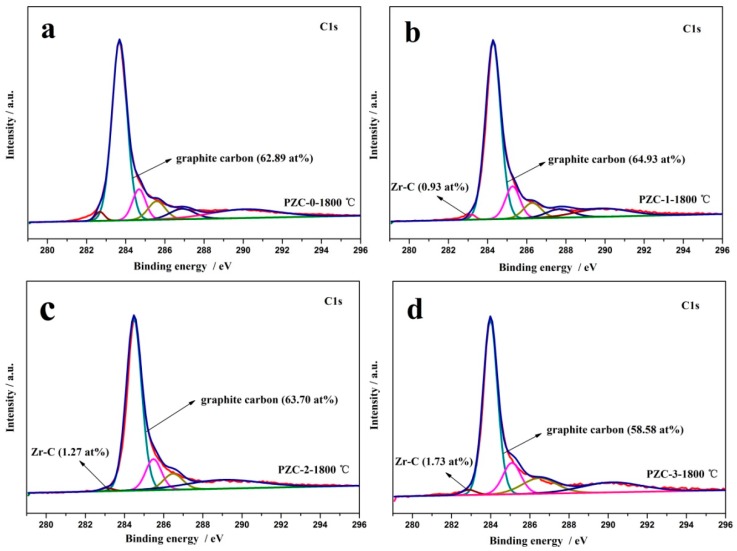
C_1s_ XPS fitting spectra of PZC-derived carbons pyrolyzed at 1800 °C: (**a**) PZC-0; (**b**) PZC-1; (**c**) PZC-2; (**d**) PZC-3.

To further explore the microstructure of the carbon phase in ZrC–C ceramics, XPS technique was applied and the result is shown in Figure 3. Different carbon constituents of all the samples are listed in Table 2. In the C_1s_ XPS of PZC-0 derived carbon, the peak centered at 283.68 eV is ascribed to C–C and C=C groups [23]. The C_1s_ XPS of PZC-*x* (*x* = 1, 2, 3) derived ceramics shows a dominating component positioned at around 284 eV, corresponding to the C=C and C–C groups, which is the energy of C_1s_ core level in the carbon species of aromatic ring [24,25]. The shift of the peak position implies the potential interaction between zirconium and the carbon phase, as confirmed by the presence of Zr–C bond. The peak at 284.7 eV is ascribed to C–C bonds representing amorphous carbon or adventitious carbon [26]. The peak at 285.6 eV is mainly from the combination of C–O groups [26,27]. The peak at 286.9 eV is the characteristic of C=O groups of aldehyde products, and the peak at 290.1 eV arises from COO carboxyl groups [26]. Moreover, the peaks centered at around 283 eV are ascribed to Zr–C bond [28]. For the carbon phase in PZC derived ceramics, the contents of the Zr–C bond are 0 at%, 0.93 at%, 1.27 at%, and 1.73 at%, respectively, representing an enhanced interaction between the carbon phase and zirconium with the increase of ZrC content. Moreover, the main constituents of the carbon phase are C–C and C=C group (62.89 at%), C–C and C=C group (64.93% at%), C–C group (sp^3^, 63.70 at%), and C–C group (sp^2^, 58.58 at%), correspondingly, which are all originated from the graphitic structure.

### 3.3. XRD Analysis of PZC Derived Carbons

**Figure 4:**
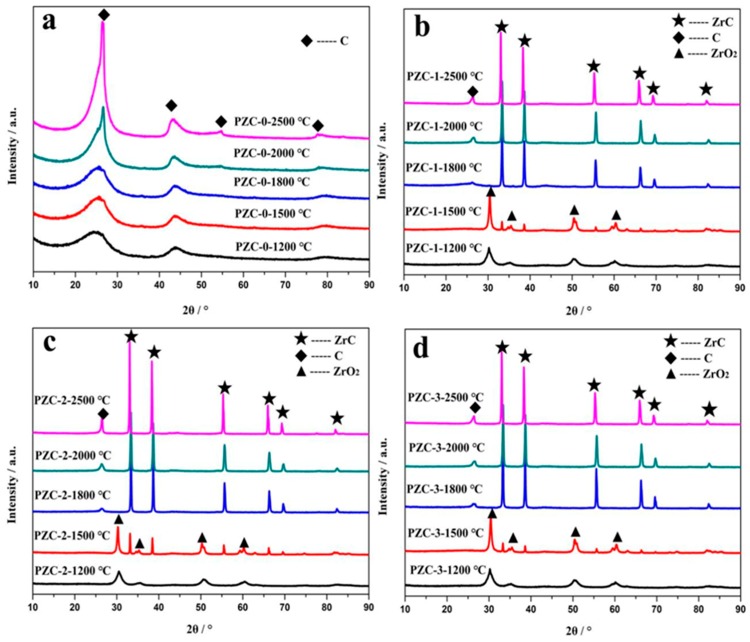
XRD patterns of PZC-derived ceramics obtained at various temperatures: (**a**) PZC-0; (**b**) PZC-1; (**c**) PZC-2; (**d**) PZC-3.

The XRD patterns of PZC heat-treated at different temperatures are given in Figure 4a–d. The corresponding d-spacings (*d*_002_), *L*_c_, 2θ angles, and full width at half maximum (HWHM) values are summarized in Table 3. For sample PZC-0 derived ceramics, only broad peaks ascribed to disordered carbons are observed, indicating the composition of the pyrolysis product is pure carbon without the existence of any ceramic phases. As the calcining temperature increases from 1200 °C to 2500 °C, the intensity of the carbon phase reflection peaks improves and the peaks become shaper, implying the increased crystallinity. For PZC-*x* (*x* = 1, 2, 3) derived ceramics, all the samples exhibit XRD patterns belong to ZrO_2_ at 1200 °C. As the calcining temperature rises to 1500 °C, weak peaks of ZrC appear, indicating that the ZrO_2_ phase begins to transform into ZrC phase. Further increasing the temperature to 1800 °C, no ZrO_2_ reflection peaks is detected. Characteristic peaks of ZrC with high intensity are observed. At the same time, the broad peaks at 2θ angles of about 26° ascribed to disordered carbon phase can be noted at this temperature. By increasing the temperature from 1800 °C to 2500 °C, the compositions of the samples remain unchanged, while the crystallinity is improved, as indicated from the increased intensities of the reflection peaks. Besides, a slight shift of ZrC peaks can be observed for all the samples obtained at 1500–2000 °C relative to that of 2500 °C. As identified by the following analysis, graphitization was processed at a higher rate in the temperature range of 1500–2000 °C, in which amorphous carbon was transformed into graphite carbon. While, further heating at 2500 °C induced the precipitation of coarse ZrC from the carbon matrix [7]. Usually, slight shift of peaks to a higher angle resulted from the doping of smaller sized element into the crystal lattice through substitution. Therefore, it can be assumed that the slight shift of ZrC peaks was caused by the dissolution of C into the crystal structure of ZrC, which may be one of the key steps for the graphitization of amorphous carbon.

Compared with PZC-0 derived carbon sample, the carbon phase in ZrC-C ceramics pyrolyzed from sample PZC-*x* (*x* = 1, 2, 3) presents a much smaller FWHM value and a much larger *L*_c_ value. For the PZC-0 derived carbon, the average *L*_c_ size grows from 21.22 nm to just 26.26 nm as the heat-treatment temperature increases from 1800 to 2500 °C. Whereas for that of PZC-3 derived carbon, the average *L*_c_ size grows from 26.25 nm to 37.51 nm under the same condition, indicating a significantly improved stacking of the layer planes with the presence of ZrC. Furthermore, decreased *d*_002_ of the carbon phase can also be found in the PZC-*x* (*x* = 1, 2, 3) derived carbons, demonstrating that zirconium may play a catalytic role to promote the increase in crystallite height and decrease in interlayer spacing of the carbon phase, which is consistent with the report concerning the catalytic graphitization of zirconium [17]. The reason can be explained as the electron deficiency of zirconium decreases the repulsive interaction between the π-electron clouds of the adjacent graphene layers, allowing the layers to be closer [29]. However, compared with PZC-1 and PZC-3-derived carbons, the FWHM and *d*_002_ are the smallest and *L*_c_ is the highest for PZC-2-derived carbon. Based on the above analysis, it can be concluded that crystallinity of the carbon phase and the crystalline growth can be significantly improved by the existence of ZrC. However, high ZrC content may show a negative effect on the stacking of the layer planes. In addition, the data shows that the increase of zirconium content exhibits an obvious effect on the value of *d*_002_, but with no distinct regularity, indicating that there may be a complex interaction between the carbon phase and zirconium, which will be further clarified in the following part.

**Figure 5:**
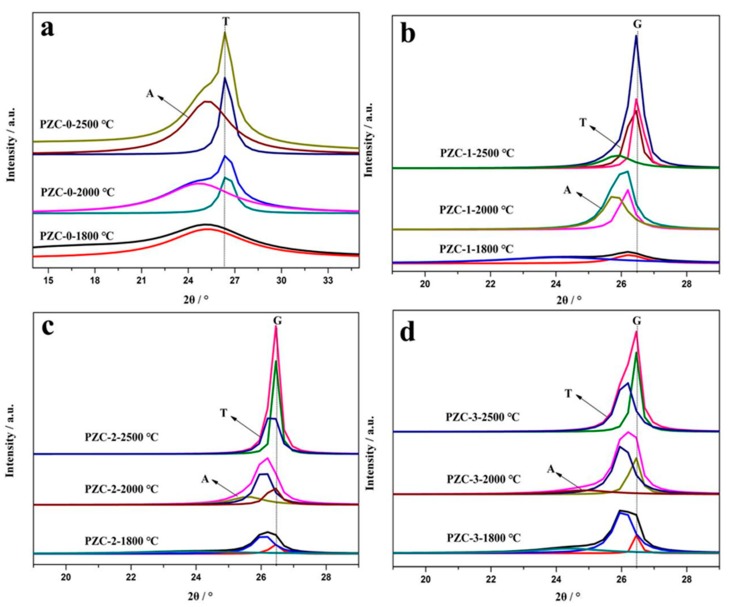
(002) Diffraction profiles of PZC-derived carbons obtained at 1800, 2000, and 2500 °C: (**a**) PZC-0; (**b**) PZC-1; (**c**) PZC-2; (**d**) PZC-3.

Usually, the (002) peak of carbon phase can be resolved into an amorphous (A), a turbostratic (T), i.e., pre-graphite, or a graphitic (G) component, i.e., the bulky graphite crystal. During the graphitization process, component A reacts with the zirconium to generate carbide first. Then component A dissolves into the carbide and subsequently reprecipitates as component G through the carbide, referred to as dissolution-reprecipitation mechanism [30]. The relative content of component G represents the graphitization effect of the catalyst. To further clarify the microstructure of the carbon phase, the (002) peaks of PZC derived carbon phases are fitted and presented in Figure 5. The relative content of component A, T, and G are calculated and shown in Table 4. As observed, the PZC-derived carbons are characterized by broad (002) peaks, indicating a high disordered carbon structure. As the heat-treatment temperature (HTT) increases, the peak becomes narrowed and shifts toward 26.5°, i.e., the (002) of the ideal graphite, indicating the improved graphitization degree. To be specific, the (002) peak of PZC-0-1800 derived carbon is resolved into an amorphous (A) component. As temperature increases, the component is confirmed to be amorphous (A) and turbostratic (T). The content of component A decreases and the content of component T tends to increase with the increasing of HTT. The (002) peak of PZC-1-1800 derived carbon is resolved into an amorphous (A) and turbostratic (T) component. With the increasing of HTT, the content of component A decreases significantly, the content of component T increases, and component G appears when HTT is 2500 °C. As for PZC-2-1800 and PZC-3-1800derived carbons, the peak can be fitted into three components, i.e., A, T, and G. The decrease in component A and a slight increase in component T and G are identified after heat-treatment at 2000 °C. Further increasing the HTT to 2500 °C, component A in the carbon phase disappears.

Compared with PZC-0-derived carbon, dramatically decrease of component A and the formation of a large amount of component T and G are noted for PZC-1-derived carbon, indicating the zirconium is crucial to enhance the graphitization process. Further increasing the zirconium content (sample PZC-2 and PZC-3), the relative contents of component T and G tend to increase accompanied by the disappearance of component A. However, a slight decrease in component T and G for PZC-3 derived carbon can be noted, implying that high zirconium content may exhibit a negative effect on the graphitization. During the graphitization process, zirconium was bonded with vicinity carbon atoms of graphite microcrystalline and tended to dissolve non-graphite carbon and precipitated graphite carbon [26]. Therefore, high amount of zirconium in contact with carbon may delay the graphitization process to some degree. Thus a slight decrease in the amount of component T and G for PZC-3 derived carbon is observed.

### 3.4. Raman Analysis of PZC-Derived Carbons

Raman spectrum of the carbonaceous materials usually shows two specific absorption peaks located in the ranges of 1340–1360 cm^−1^ called D-band and 1580–1600 cm^−1^ called G-band, respectively. The D-band corresponds to short-range turbostratic structures, and the G-band represents the normal graphite structure [31]. For materials with higher degree of structure order, 2D peak appears at about 2700 cm^−1^. The positions of the G and 2D peaks, the microcrystalline planar size (*L*_a_), *I*_D_/*I*_G_ ration (R), and the full width at half maximum (FWHM) are commonly used to characterize the structure of carbon materials [32,33].

**Figure 6:**
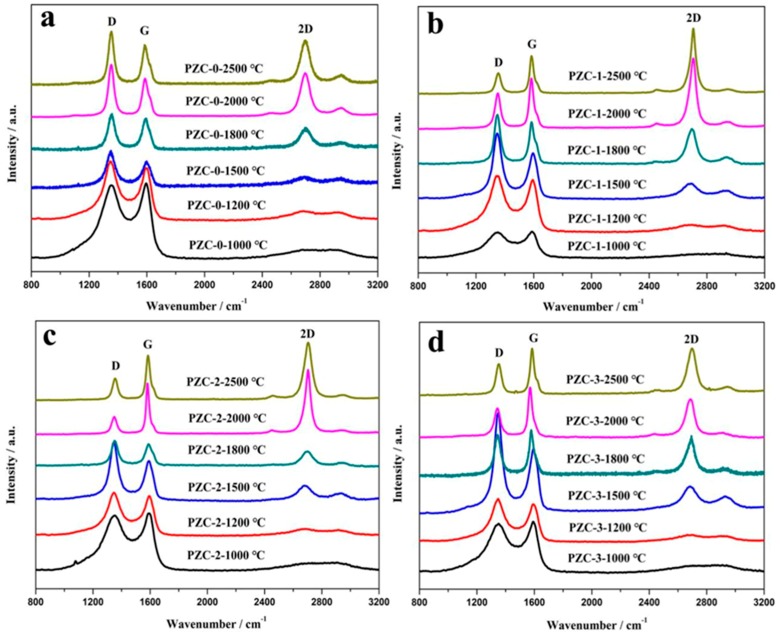
Raman spectra of PZC-derived carbons pyrolyzed at various temperatures: (**a**) PZC-0; (**b**) PZC-1; (**c**) PZC-2; (**d**) PZC-3.

**Figure 7:**
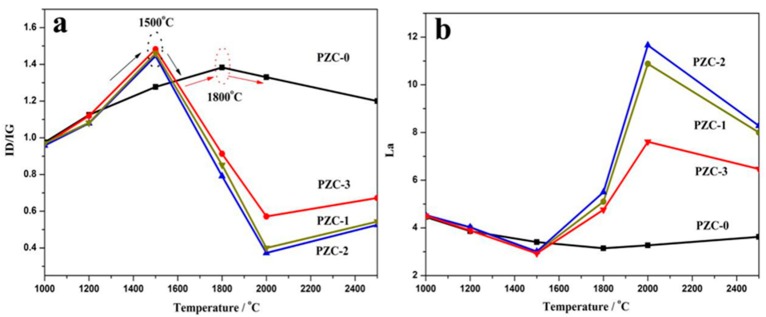
Raman spectra parameters of PZC-derived carbons pyrolyzed at various temperatures: (**a**) ID/IG; (**b**) La3.5. Mechanism discussion.

Figure 6 shows the Raman spectra of PZC-derived carbons over the wave number range of 800–3200 cm^−1^. The relationships of the *I*_D_/*I*_G_ and *L*_a_ values versus HTT are shown in Figure 7. With the increase of HTT, the D peak and G peak become shaper, and the 2D peak appears and becomes narrowed for each sample. Compared with sample PZC-0, *I*_D_/*I*_G_ value has dropped more than half, and *L*_a_ is doubled for PZC-*x* (*x* = 1, 2, 3) derived carbons. These changes demonstrate that the graphite structure is significantly improved because of the existence of zirconium. For PZC-0-derived carbon, *I*_D_/*I*_G_ increases and *L*_a_ decreases in the temperature range of 1000 to 1800 °C. Subsequently, *I*_D_/*I*_G_ decreases and *L*_a_ increases at temperatures higher than 1800 °C. For PZC-*x* (*x* = 1, 2, 3) derived carbons, the temperature of structure improving turning point is reduced from 1800 °C for PZC-0-derived carbon to 1500 °C, which was the initial formation temperature of ZrC. Further increasing the temperature from 1500 to 2000 °C, *I*_D_/*I*_G_ decreases and *L*_a_ increases at a much higher rate, compared with that of PZC-0. This result demonstrates that the formation of ZrC plays a crucial role in promoting the graphitization of the carbon phase. In the temperature of 2000–2500 °C, as the ZrC particles grow, *I*_D_/*I*_G_ increases and *L*_a_ decreases, indicating a reduced structure order at this stage.

As is known, the pyrolyzed carbon tends to form a more rigid and inflexible structure for non-graphitizing carbon. This structure may prevent the graphitic layers arrangement, thus restrains the graphitization process [34]. Based on the XRD analysis, 1500 °C is the initial formation temperature of ZrC ceramic through the carbothermal reduction reaction between the carbon phase and ZrO_2_. Since the inherent broken structural symmetries pyrolyzed from a three-dimensional cross-linked structure in the precursor polymer is considered as the underlaying reason for the non-graphitizing behavior [35,36], it can be deduced that the carbothermal reaction which may break the crosslinked carbon structure is the key step to accelerate the graphitization process in our experiment. According to our previous study concerning the pyrolysis process of the precursor [7], further increasing temperature to 2500 °C leads to the remarkable grain growth and precipitation of ZrC particles, i.e., coarsening and aggregation of ZrC particles. This process may destroy the surrounded ordered carbon structure and cause the generation of defects in the carbon phase, leading to larger *I*_D_/*I*_G_ and smaller *L*_a_ for the sample pyrolyzed at 2500 °C. Moreover, among PZC-*x* (*x* = 1, 2, 3) derived carbons, *I*_D_/*I*_G_ is found to be the lowest and *L*_a_ is the largest for sample PZC-2-derived carbon, indicating that high zirconium content exhibits negative effects on the structure order to a certain extent. This result is consist with the analysis of XPS and XRD.

**Figure 8:**
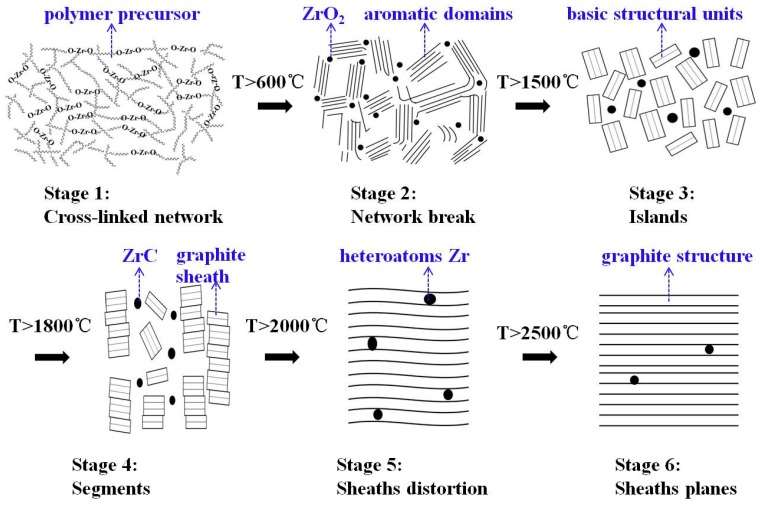
Schematic steps of graphitization and sketches of carbon phase in PZC-derived ZrC–C ceramics.

Combining the graphitization process proposed by Rouzaud and Oberlin [37] with the above analysis, the effects of the ZrC formation on graphitization of the carbon phase in ZrC–C ceramics are described as follows (shown in Figure 8). For stage one (raw precursor), based on our previous study, the incorporation of Zr– on to the polymer backbone was confirmed, and the prepared PZC precursor might be Zr–O and Zr–O–C chain polymer with acetylacetone and hydroxymethyl phenol as ligands [7]. Therefore, the zirconium elements are uniformly distributed in the form of Zr–O bonds. In the second stage (higher than 600 °C), the network of the polymer collapses with the formation of ZrO_2_ particles and aromatic domains. ZrO_2_ particles are trapped within the pyrolysis product of the precursor. With the increasing of HTT, pyrolysis of the polymer is enhanced, accompanied by the generation of non-graphitized carbon, as well as the increase of ZrO_2_ crystallinity. In the third stage (higher than 1500 °C), the temperature of which is the initial temperature of carbothermal reduction reaction between ZrO_2_ and C to form ZrC, ZrO_2_ crystalline is segregated drastically by these aromatic domains, similarly to segregated “island” structure, deemed as basic structural units (BSU). Since the as-prepared precursor is a three-dimensional cross-linked hybrid structure consisting of aromatic clusters, the pyrolysis carbon obtained belongs to non-graphitizable hard carbon. The thermal stable structure of aromatic clusters connected by tetrahedral C–C bonds, which restricts the movement of BSU, is believed to be the key barrier that blocks the process to form highly-ordered graphite. During this stage, carbothermal reduction reaction breaks the tetrahedral C–C bonds, thereby reducing the barrier to graphitization in the amorphous carbon. Thus the graphitization process was significantly promoted as a result of the ZrC formation. When the HTT is higher than the initial temperature of carbothermal reduction reaction, the graphitization is significantly enhanced because of the increased BSU movement. For the fourth stage (higher than 1800 °C), the graphitization proceeds according to the dissolution-reprecipitation mechanism [38]. Based on the phase diagram, the carbon solubility is high in the ZrC–C system at high temperatures, as reported that carbon solubility is 20.2 at% at the melt temperature [39]. Non-graphite carbon component dissolves into the nearby ZrC, and then subsequently reprecipitates as the graphite carbon component. With the increase of HTT, more carbon will dissolve into ZrC particles, the dissolution-reprecipitation sequence occurs through the carbide and finally reaches to a dissolution-reprecipitation dynamic equilibrium. The reprecipitation of graphite carbon enhances the growth of graphite sheath, which allows the generation of imperfectly stacked BSU columns. In the following stages (higher than 2000 °C), inter-layer defects and heteroatoms Zr are annealed out of the graphite structure. ZrC particles reprecipitate by breaking the linkage with vicinity carbon atoms of the graphite microcrystalline, which will bring defects into the graphite structure to some extent. Moreover, the coarsening and aggregation of ZrC particles during the heat-treatment will distort the graphite planes (shown in stage five). These may explain the reason for the slightly lowered structure order for PZC-3 derived ceramics with a higher ZrC content. Further increasing the heat-treatment temperature higher than 2500 °C as shown in stage 6, ZrC will be removed out of the graphite structure, and the defects in the graphite will be healed, thus forming highly crystalline graphite.

## 4. Conclusions

To clarify the effect of ZrC formation on the evolution of the carbon phase, microstructure of the carbon phase during the ceramization process of polymer precursor was investigated. For the carbon phase in ZrC-C ceramics, an significantly enhanced degree of crystal growth was observed. With the increase of ZrC content, amorphous (A) component in the carbon phase disappeared, while the content of turbostratic (T) component and graphitic (G) component was increased. Moreover, ZrC content showed no obvious effect on the relative content of component T and G. During the heat-treatment, carbothermal reduction reaction between the carbon phase and ZrO_2_ to form ZrC ceramic is crucial to break the stable structure of aromatic clusters, thereby reducing the barrier of the graphitization in the amorphous carbon, thus significantly improving the graphitization process. The minimum *I*_D_/*I*_G_ (0.77) and *d*_002_ (0.3383 nm), maximum *L*_a_ (5.64 nm) and *L*_c_ (37.51 nm) were obtained for PZC derived carbon. However, a high amount of zirconium will restrain the graphite crystalline growth to a certain extent. Also, coarsening and aggregation of the ZrC particles resulted from high-temperature heat-treatment would exhibit negative influence on the structure order.

## Figures and Tables

**Table 1:** Zirconium content in PZC precursors and the derived ceramics.

Sample	ZrOCl_2_·8H_2_O content/g ^a^	A(Zr-O)/A(C=C)	Zirconium Content/wt.% ^b^	Zirconium Content/wt.% ^c^
PZC-0	0	0	0	0
PZC-1	5	0.64	5	11
PZC-2	10	0.66	10	18
PZC-3	15	0.68	16	27

(a) Weight of ZrOCl_2_·8H_2_O in feed. (b) Approximate zirconium mass fraction in PZC precursors based on the XPS analysis. (c) Approximate zirconium mass fraction in ZrC–C ceramics annealed at 1800 °C based on the EDS analysis.

**Table 2:** Constituents of PZC-derived carbons pyrolyzed at 1800 °C analyzed from C_1s_ XPS.

Sample	Peak Position (eV)	at% of Total Area	Carbon Constituents
PZC-0	283.68	62.89	C–C, C=C
	284.68	9.43	Polymeric C–C
	285~290	27.68	C–O, C=O
PZC-1	283.18	0.93	Zr–C
	284.28	64.93	C–C, C=C
	285–290	34.05	C–O, C=O
PZC-2	283.08	1.27	Zr–C
	284.48	63.70	C–C (sp^3^)
	285–290	35.03	C–O, C=O
PZC-3	282.88	1.73	Zr–C
	283.98	58.58	C–C (sp^2^)
	285–290	39.68	C–O, C=O

**Table 3:** Parameters of (002) diffraction peaks of PZC-derived carbons obtained at 1800, 2000, and 2500 °C.

Heat-Treatment Temperature (HTT, °C)	Sample	2θ (002)	HWHM_002_ (°)	*d*_002_ (nm)	*L*_c_ (nm)
1800	PZC-0	26.56	0.4018	0.3403	21.22
	PZC-1	26.23	0.3247	0.3395	26.25
	PZC-2	26.43	0.3247	0.3370	26.26
	PZC-3	26.23	0.3247	0.3396	26.25
2000	PZC-0	26.73	0.3572	0.3349	23.88
	PZC-1	25.58	0.2982	0.3480	28.54
	PZC-2	26.38	0.2598	0.3375	32.81
	PZC-3	26.78	0.2922	0.3329	29.20
2500	PZC-0	26.43	0.3247	0.3370	26.26
	PZC-1	26.38	0.2923	0.3332	29.16
	PZC-2	26.60	0.2273	0.3348	37.52
	PZC-3	26.46	0.2273	0.3366	37.51

**Table 4:** Components for PZC derived carbons obtained at 1800, 2000, and 2500 °C.

Sample	Components (%) (HTT, 1800 °C)			Components (%) (HTT, 2000 °C)			Components (%) (HTT, 2500 °C)		
	A	T	G	A	T	G	A	T	G
PZC-0	1	0	0	0.846	0.157	0	0.709	0.291	0
PZC-1	0.734	0.262	0	0.601	0.399	0	0.214	0.412	0.375
PZC-2	0.358	0.509	0.133	0.281	0.511	0.208	0	0.518	0.482
PZC-3	0.250	0.668	0.083	0.144	0.601	0.255	0	0.592	0.408
